# Doppler effect induced spin relaxation boom

**DOI:** 10.1038/srep23169

**Published:** 2016-03-21

**Authors:** Xinyu Zhao, Peihao Huang, Xuedong Hu

**Affiliations:** 1 Department of Physics, University at Buffalo, SUNY, Buffalo, New York 14260, USA; 2 Quantum Measurement Division, National Institute of Standards and Technology, Gaithersburg, MD, 20899-8423, USA.; 3 Joint Quantum Institute, University of Maryland, College Park, MD, 20742, USA.

## Abstract

We study an electron spin qubit confined in a moving quantum dot (QD), with our attention on both spin relaxation, and the product of spin relaxation, the emitted phonons. We find that Doppler effect leads to several interesting phenomena. In particular, spin relaxation rate peaks when the QD motion is in the transonic regime, which we term a spin relaxation boom in analogy to the classical sonic boom. This peak indicates that a moving spin qubit may have even lower relaxation rate than a static qubit, pointing at the possibility of coherence-preserving transport for a spin qubit. We also find that the emitted phonons become strongly directional and narrow in their frequency range as the qubit reaches the supersonic regime, similar to Cherenkov radiation. In other words, fast moving excited spin qubits can act as a source of non-classical phonons. Compared to classical Cherenkov radiation, we show that quantum dot confinement produces a small but important correction on the Cherenkov angle. Taking together, these results have important implications to both spin-based quantum information processing and coherent phonon dynamics in semiconductor nanostructures.

The allure of ultra-powerful quantum computers has pushed for ever more precise knowledge of quantum coherent dynamics in semiconductor and superconductor nanostructures. The acquired understanding may also lead to technological applications now, as best illustrated by extremely sensitive magnetic field sensors made from Nitrogen-Vacancy centers in diamond. Electron spin qubit is a promising candidate for realizing quantum computing because of its long coherence time^1^,^2^,^3^,^4^. It has attracted extensive research interests over the past decade, with studies mostly focusing on the fabrication and manipulation of spin qubits confined in a fixed quantum dot or dopant ion^5^,^6^,^7^.

In a large-scale quantum computer, quantum information needs to be transferred over finite distances frequently. For electron-spin-based qubits, one straightforward way to achieve such communication is to move the confined electrons themselves directly. There are several proposed schemes on how to move the spin qubits efficiently^8^,^9^,^10^,^11^,^12^,^13^,^14^,^15^,^16^,^17^, where a controlled motion of the confined electron can be induced by either varying gate voltages or a surface acoustic wave (SAW). However, introducing this orbital (albeit controlled) dynamics inevitably weakens the orbital quantization that gives rise to the long electron spin coherence times^1^,^2^,^3^,^4^. Considering that spin-orbit interaction (SOI) together with electron-phonon interaction is the dominant spin relaxation channel in a fixed quantum dot^1^, and SOI is the main reason for spin mixing and relaxation in bulk semiconductors^18^, study spin relaxation for a moving electron spin qubit is crucial in establishing the viability of this spin communication approach.

A moving spin qubit in the excited spin state can be thought of as a phonon source. An examination of its relaxation should also include analysis of the features of the emitted phonon. Classically, such a moving source of a wave experiences the Doppler effect, which refers to a frequency shift of the emitted wave due to relative motion between the source and an observer. Doppler effect leads to a range of interesting physical phenomena. A commonly observed example is when an observer hears different pitches from the horn of an approaching or a departing vehicle. When the speed of the source is larger than the speed of the waves it produces, a directional shock wave (Cherenkov effect) can be observed, as in the wake of a speeding boat, the sonic boom from a supersonic airplane, and the Cherenkov radiation from a fast-moving charge in a material with high refraction index^19^,^20^,^21^,^22^ (as depicted in Fig. 1). An intriguing question here is whether these classical features would translate into a quantum system of a moving but confined electron, and how they may be modified by quantum mechanics. If the emitted phonons indeed have strongly peaked directional and spectral properties, the moving electron spins could potentially be candidates as a source of non-classical phonons.

Here we study how the motion of a spin qubit modifies its relaxation due to SOI and electron-phonon interaction^23^,^24^,^25^,^26^. We identify different regimes of quantum dot moving velocity where we find analogues of Doppler effect, sonic boom in the spin relaxation, Cherenkov radiation, and the associated phonon emission. Specifically, we find that when the quantum dot (QD) moves with a speed lower than the speed of sound, the energy of the emitted phonon during spin relaxation is dependent on the direction of emission, similar to the Doppler effect. When the QD moves faster than the speed of sound, the dominant contributions to spin relaxation come from phonons emitted along certain directions, similar to the classical Cherenkov effect, but with quantum dot confinement for the electron making a correction to the Cherenkov angle. In the transonic regime, we observe a peak in spin relaxation rate, which we term as a spin relaxation boom in analogy to the classical sonic boom. In addition, our study indicates that the emitted phonon by the moving spin qubit is highly directional and narrow in its frequency distribution. Thus a stream of such excited spin qubits could act as a source for highly non-classical phonons in a semiconductor nanostructure.

## Results

### Model and solution

**Figure 1:**
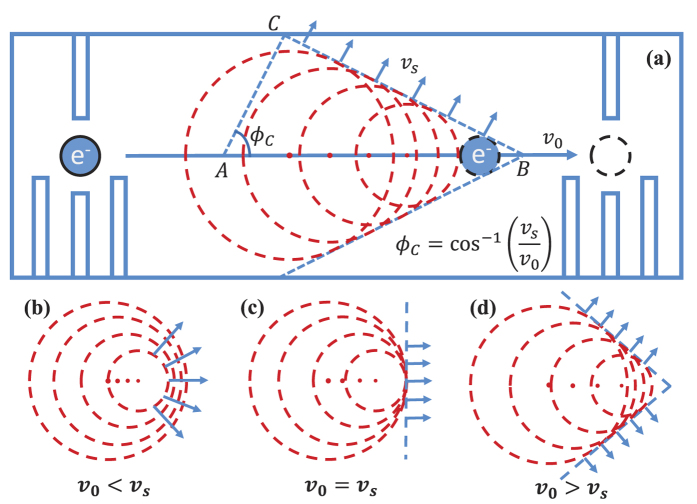
A schematic diagram of a moving spin qubit interacting with phonon reservoir and the resultant Doppler effect in the three cases.

The system we consider is a single electron confined in a moving QD formed from a two-dimensional electron gas (2DEG), as is shown in Fig. 1. The qubit (electron) is moved at a constant speed *v*_0_, presumably achieved by programming the gates or using the surface acoustic waves. Conceptually, to ensure such a uniform linear motion for the electron, there has to be an external driving force, which we treat as classical. The total Hamiltonian^17^,^23^,^25^ is given by


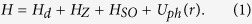


Here 
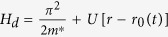
 is the orbital Hamiltonian for the moving QD, where 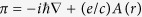 is the 2D momentum operator of the electron, and m^*^ is the effective mass of the electron. The motion we considered is linear: *r*_0_(*t*) = *v*_0_*t*, and the confinement potential 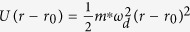 is quadratic. 
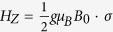
 is the Zeeman Hamiltonian, with *B*_0_ the applied magnetic field. *H*_*SO*_ = *β*_−_*π*_*y*_*σ*_*x*_ + *β*_+_*π*_*x*_*σ*_*y*_ is the spin-orbit (SO) interaction, where *β*_±_ = *β* ± *α* give the SO coupling strength, with *α* and *β* being the strengths of Rashba^27^ and Dresselhaous^28^ SO interaction, respectively. Lastly, the electron-phonon interaction is given by^23^,^25^,^29^


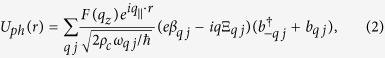


where 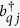 and *b*_*q j*_ are the creation and annihilation operators for an acoustic phonon with wave vector 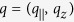 and branch index *j*, and *ρ*_*c*_ is the density of the material. The function 
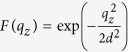
 describes the confinement along the *z* direction, with *d* the characteristic width of the quantum well. We take into account both piezoelectric coupling (*β*_*q j*_) and deformation potential (Ξ_*q j*_) coupling in the electron-phonon interaction^29^,^30^. By performing a Schrieffer-Wolff transformation to remove the SO coupling term to the first order^17^,^23^,^24^,^25^,^31^, the effective spin Hamiltonian can be obtained as


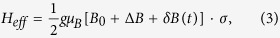


where 
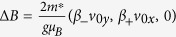
 is a motion induced constant magnetic field for the spin, and 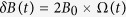 is the motion-induced magnetic noise from the phonons, where Ω(*t*) originates from the electric noise due to the phonons


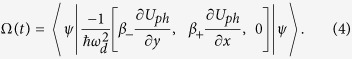


Here 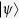 is the instantaneous orbital ground state of the QD, so that 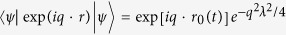, where 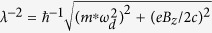 is the total confinement length of the QD.

With the effective Hamiltonian (3), and assuming the phonons are in thermal equilibrium, the spin relaxation rate can be obtained as


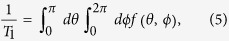


where the kernel function (or angular distribution function) takes the form


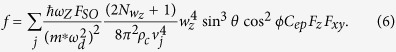


Here


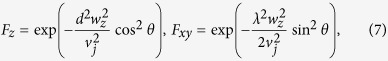


are the cutoff functions in the *z* direction and the *xy* plane, respectively, due to the quantum dot confinement potential. The constant 
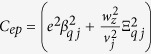
 gives the total strength of the two types of electron-phonon interaction, namely the deformation potential coupling and the piezoelectric coupling. *v*_*j*_ are the speed of sound with branch index *j*. 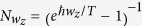 is the number of phonons with frequency *w*_*z*_ at thermal equilibrium. The factor *F*_*SO*_ in equation (6) describes the angular dependence of the magnetic noise on the direction of the applied field due to SOI. Its explicit expression is *F*_*SO*_ = (*β*^2^ + *α*^2^)(1 + cos^2^ *θ*_*B*_) + 2*αβ* sin^2^ *θ*_*B*_cos(2*φ*_*B*_). Lastly, the kernel function *f*, and therefore the spin relaxation rate 1/T_1_, depends on a direction-dependent “shifted frequency” for the phonons,


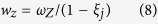


where 
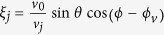
. Clearly, this frequency is different from the spin Zeeman splitting 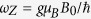, because of the Doppler shift for the phonon field experienced by the moving spin qubit. It is worth noting that equation (8) is always combined with a condition 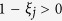 to avoid negative “shifted frequency” which is unphysical. Actually, a detailed mathematical derivation in section Methods also proves that those negative frequencies are indeed excluded from the integration in equation (5).

In this calculation, spin relaxation is caused by the interaction between the electron and the lattice phonons from all directions. The double integration over *θ* and *ϕ* in equation (5) is from the summation 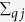 over all the phonon wave vectors *q* in equation (2). The kernel function 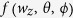 thus describes contributions by phonons emitted or absorbed in the infinitesimal solid angle *dθdϕ* around (*θ*, *ϕ*).

In our numerical calculations, we use typical parameters in a GaAs QD. There is one branch of longitudinal acoustic (LA) phonons, and two branches of transverse acoustic (TA) phonons. *v*_1_ = 4730 m/s is the sound speed of the LA phonons, while *v*_2_ = *v*_3_ = 3350 m/s are the sound speed of the TA phonons. The strength of the deformation potential is Ξ_1_ = 6.7 eV. The strengths of the piezoelectric interaction are 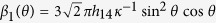, 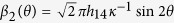, and 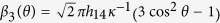, where 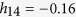 C/m^2^ and *κ* = 13.1^23^.

With the help of the analytical expression of the relaxation rate, we could examine the angular characteristics of the emitted phonons in term of the kernel function *f* and the total relaxation rate 1/*T*_1_ for the moving spin qubit.

### Directional Phonon Emission: Doppler effect and Cherenkov Radiation

In this subsection we analyze the angular dependence of the phonon emission (in terms of the kernel function *f*  ) from the relaxing spin qubit in different regimes of QD moving speed. In particular, in the subsonic regime, we find the Doppler effect, in which phonons emitted in different directions have different frequencies. In the transonic regime we find the formation of a shock wave front and its bifurcation into two directions as the QD speed passes the speed of sound. Lastly in the supersonic regime we find a phonon analog of Cherenkov radiation, and identify a quantum dot confinement induced correction in the Cherenkov angle.

### Doppler effect

When a QD with one confined spin qubit moves relative to the lattice with a speed smaller than the speed of sound, the frequency of the phonon emitted or absorbed is shifted with a Doppler factor 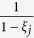, as indicated in equation (8). In particular, in the forward direction (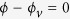 and *θ* = *π*/2), an emitted phonon has an increased frequency *ω*_*Z*_/(1 − *v*_0_/*v*_*j*_), while in the backward direction the phonon frequency is reduced to *ω*_*Z*_/(1 + *v*_0_/*v*_*j*_). These shifts are exactly as one would find in the classical Doppler effect.

It may seem puzzling that the energy quantum carried by the emitted phonon is not the same as the Zeeman splitting of the spin qubit. The discrepancy here is because of the fact that the moving quantum dot is an open system. It is driven by a classical force that comes from either programmed gate potential or the large number of phonons in an SAW. The excess or shortage of energy in the spin relaxation is absorbed/added by this classical “reservoir”.

### Breaking the sound barrier

If the moving spin qubit can be regarded as a classical phonon emitter (or sound emitter), the transition from subsonic regime to supersonic regime (the transonic regime) for the moving spin qubit would be well represented by Fig. 1(b–d). At low speeds, presented in panel (b), there is no strongly directional emission. When the QD moving velocity becomes equal to the sound velocity, as indicated in panel (c), a single forward-propagating shock wave front is formed. When the moving velocity is larger than the critical velocity (d), the single shock wave front splits into two (We only plot the cross section in the *xy* plane. In three-dimension the wave front is conical).

**Figure 2:**
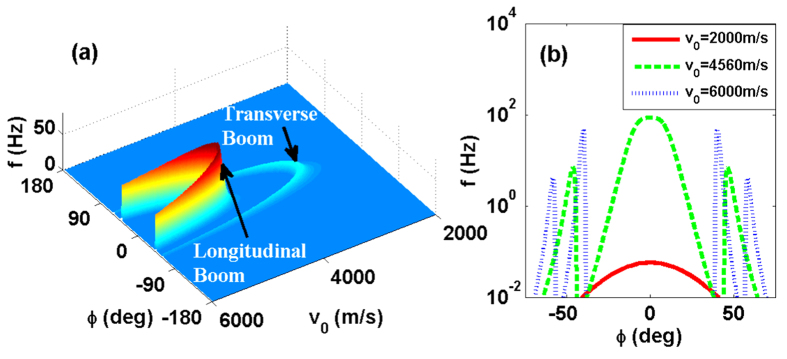
(**a**) Angular distribution (azimuthal angle *ϕ*) of the kernel function 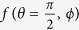 for different moving velocity *v*_0_. Here the polar angle *θ* is fixed at 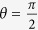. The parameters are *B*_0_ = 1 T, *ω*_*d*_ = 10.1 meV, *d* = 20 nm, *ϕ*_*v*_ = 0. (**b**) Three cross-sections of (**a**) at different velocities. The red solid line has a velocity below the speed of the transverse acoustic phonons; the green dashed line has a velocity at the speed of longitudinal acoustic phonons, and the blue dotted line has a velocity above the speed of longitudinal phonons.

The moving spin qubit is a highly quantum mechanical object, emitting or absorbing only a single phonon at a time. Nevertheless, we find that it displays a behavior qualitatively similar to a classical phonon source. For example, Fig. 2(a) shows the QD speed *v*_0_ and angle *ϕ* dependence of the kernel function *f* qualitatively (we have chosen *θ* = *π*/2 to maximize *f*). When *v*_0_ < *v*_1_, the angular distribution is well described by SOI, with a pretty strong but smooth sin^3^*θ*cos^2^*ϕ* angular dependence for *f*, so that emission along directions perpendicular to the direction of motion is suppressed, while emissions along all other directions are allowed. When *v*_0_ ≈ *v*_1_, the angular distribution in the *xy* plane rapidly becomes concentrated around *ϕ* = 0°, as *ϕ* = 0° is a singularity of *w*_*z*_ when *v*_0_ = *v*_1_. Finally, when *v*_0_ > *v*_1_, the angular distribution in the *xy* plane is split into two branches. Each branch corresponds to an angle *ϕ* that gives the peak value of the kernel function *f*. As the moving velocity increases from the subsonic regime to the supersonic regime, the kernel function rapidly concentrates into the two bifurcating angles.

The transitions through the transonic regime can be more quantitatively seen from the cross sections given in Fig. 2(b). For *v*_0_ = 2000 m/s, which is subsonic, the kernel function is smooth and has a small magnitude (notice the vertical scale is logarithmic). At *v*_0_ = 4560 m/s, the speed of sound for the LA phonons, a large peak appears at *ϕ* = 0. The logarithmic scale has made this peak appears to be broader than it really is (the scale is necessary for us to see the subsonic value of *f* ). The two side peaks are the shock waves produced by the TA phonons, for whom the moving dot is already supersonic. Lastly, at *v*_0_ = 6000 m/s, the moving qubit is supersonic with respect to both LA and TA phonons. Thus two sets of shock wave peaks appear for the kernel function in this case. For the parameters used in this paper, deformation potential coupling to LA phonons provides the dominant spin relaxation channel. Therefore the critical velocity for obvious Cherenkov effect is approximately *v*_1_. For a case when piezoelectric potential is dominant or when both interactions are important, there would be two critical velocities, determined by the two types of phonons.

### The Cherenkov effect and its quantum correction

As we have discussed in the Introduction, Cherenkov radiation is a well known effect in classical physics. It refers to the formation of shock wave fronts when a wave-emitting source moves faster than the speed of the emitted wave in a media. Examples include the wake of a fast-moving boat, and directional photons emitted by a moving charge in a media of high refraction index when the charge moves faster than the phase speed of light in the media. Since a moving spin qubit in the excited state acts as a source of sound waves, we are inspired to examine whether Cherenkov effect also exists in a QD that moves faster than the speed of sound.

Our analysis of phonon angular distribution starts from the kernel function *f* for the spin relaxation rate in equation (6). For a gated QD in a semiconductor nanostructure (such as a gated depletion quantum dot from a two-dimensional electron gas), the confinement along the *z* direction (growth direction) is generally much stronger than those in the *xy* directions (in-plane directions). Consequently, in a spin relaxation process induced by spin-orbit interaction and electron-phonon coupling, phonons are mostly emitted in the *xy* plane. In the following qualitative discussion we will thus focus on the phonon emission in the *xy* plane with *θ* = *π*/2 (in our numerical calculation we do include phonons in all directions), when the kernel function *f* from equation (6) is reduced to


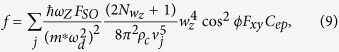


where 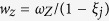 and 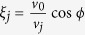 (the azimuthal direction of the quantum dot motion has been chosen to be *ϕ*_*v*_ = 0). Now *f* depends on the phonon frequency *w*_*z*_ as 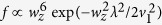 when we limit our consideration to the deformation potential interaction with LA phonons. The discussion is similar when piezoelectric interaction dominates. Clearly, the kernel function *f* is strongly peaked, with the peak phonon emission probability at frequency


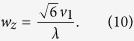


Recall that the phonon frequency and phonon direction are related because of Doppler shift, 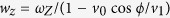, it is straightforward to obtain the peak phonon radiation angle at


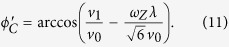


In other words, the angular distribution of the emitted phonon from a moving excited spin qubit is not uniform, but has peaks at the angles 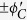. Such a strong angular concentration is a typical characteristic of the Cherenkov effect.

**Figure 3:**
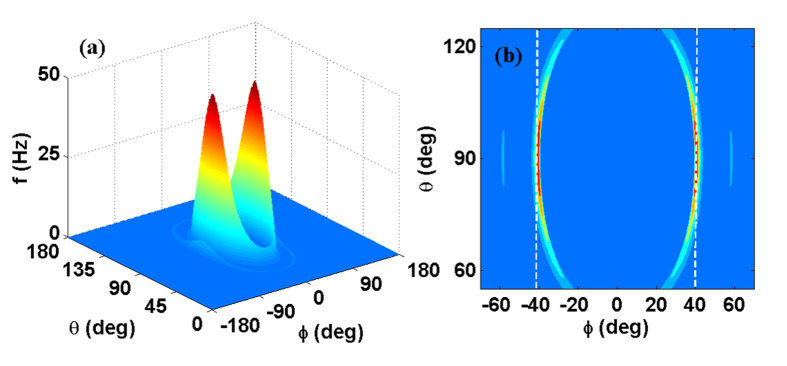
(**a**) Angular distribution of the kernel function *f*(*θ*, *ϕ*). (**b**) Contour plot of *f*(*θ*, *ϕ*) in a small region. The parameters are chosen as *v*_0_ = 6000 m/s, *B*_0_ = 1 T, *ω*_*d*_ = 0.01 eV, *d* = 20 nm, *ϕ*_*v*_ = 0.

In Fig. 3, we plot the kernel function *f* as a function of polar angle *θ* (with growth direction defined as *θ* = 0) and azimuthal angle *ϕ* when the QD speed is *v*_0_ = 6000 m/s, larger than the speed of sound for both LA (*v*_1_) and TA (*v*_2_) phonons. Clearly, the phonons making dominant contribution to spin relaxation are concentrated in two particular directions in the *xy* plane (at *ϕ* ≈ ±40° and *θ* ≈ 90°. These peaks come from the emission of LA phonons. Two much smaller peaks appear near *ϕ* ≈ ±60° and *θ* ≈ 90°), which originates from piezoelectric interaction with TA phonons. The strongly peaked angular distribution features bear a strong resemblance to the classical Cherenkov radiation.

With the parameters used in Fig. 3, equation (11) predicts a theoretical Cherenkov angle of 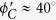. This is consistent with the numerical results [obtained from equation (6)] shown in Fig. 3, where the peak in the kernel function *f* for LA phonons appears at *ϕ* ≈ 40° for *θ* = 90°. The peak value of *f* occurs at 
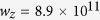
 s^−1^ when the Zeeman angular frequency *ω*_*Z*_ is 3.87 × 10^10^ s^−1^ (at *B* = 1 T). With 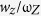 ≈ 23, the phonon energy has been Doppler-shifted greatly, from about 25 *μ*eV for *ħω*_*Z*_ to nearly 600 *μ*eV for *ħw*_*z*_.

The Cherenkov angle predicted by equation (11) is different from the prediction by classical theory. In a classical theory, based on the mechanism shown in Fig. 1, the Cherenkov angle in the *xy*-plane (*θ* = 90°) should be


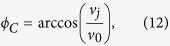


which comes out to be about 38° for LA phonons, slightly smaller than our analytical and numerical results. Equation (11) shows that a complete quantum calculation adds a correction term 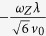 to the classical Cherenkov angle. This correction is caused by the quantum dot confinement in the form of the cutoff functions *F*_*z*_ and *F*_*xy*_ in equation (7). Without the cutoff functions, namely if we set *F*_*xy*_ = *F*_*z*_ = 1, the kernel function *f* would become monotonic 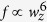. In the meantime, the classical Cherenkov radiation at 
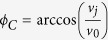
 would make *ξ*_*j*_ = 1, so that the corresponding frequency of the emitted phonons is at the unphysical limit of *w*_*z*_ → ∞. The presence of the quantum dot confinement, in the form of the cutoff functions, removes this unphysical divergence of phonon frequency, while still preserving a highly directional/anisotropic phonon emission probability distribution. Therefore, even though the modification to the Cherenkov angle is quite small (from 38° classically to 40° quantum mechanically), the inclusion of quantum dot confinement in the calculation is crucial for a physically sound description.

In short, the results presented in Fig. 3 demonstrate a quantum-corrected Cherenkov radiation of phonons from a moving spin qubit. The quantum dot confinement for the QD suppresses the electron interaction with higher-energy phonons, leading to a small but important correction to the Cherenkov radiation angle and a significant modification to the energy of the radiated phonons.

Overall, our detailed analysis of the angular distribution of the emitted phonons by the moving spin qubit shows that Doppler effect plays a significant role in shaping the characteristics of these phonons. In the subsonic regime, they are modified from when the qubit is fixed in the lattice, but the changes are quantitative instead of qualitative. As the moving speed of the qubit exceeds the speed of sound, these phonons become highly directional and narrow in their frequency distribution.

### Spin Relaxation in a Moving Quantum Dot

In the last subsection we have examined the angular behavior of phonon emission in the relaxation of a moving spin qubit. In this subsection we focus on the integrated effect of QD motion on spin relaxation. We are particularly interested in how spin relaxation varies with the speed of the QD motion and the applied magnetic field.

**Figure 4:**
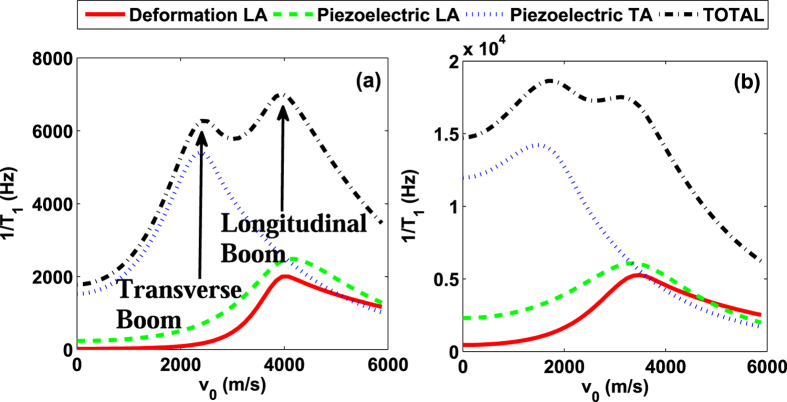
Spin relaxation rate 1/*T*_1_ as a function of moving velocity *v*_0_. The red (solid), green (dashed), blue (dotted), and black (dot-dashed) lines represent the deformation, the longitudinal piezoelectric, the transverse piezoelectric and the total spin relaxation rate respectively. The magnetic fields are *B*_0_ = 1.5 T for (**a**) and *B*_0_ = 3.5 T for (**b**). The other parameters are chosen as *ω*_*d*_ = 1.1 meV, *d* = 20 nm, *ϕ*_*v*_ = 0.

Classically, the drag force on an aircraft increases sharply when the aircraft velocity approaches the sound barrier. This is the so-called sonic boom. We find a similar behavior in the relaxation rate for a moving spin qubit. In Fig. 4, we plot the spin relaxation rate 1/*T*_1_ as a function of the QD speed *v*_0_. The curve of the total relaxation rate (black, dot-dashed) peaks at the two sound barriers due to TA (at *v*_2_ = *v*_3_) and LA phonons (at *v*_1_). Each peak for a single type of phonons (for example, the blue curve in Fig. 4(a) for the TA phonons) is similar to the Prandtl-Glauert singularity for the classical “sonic boom”. Quantum dot confinement modifies these peaks by eliminating the singularities and broadening them. The peak positions are also shifted downward from *v*_1_ and *v*_2_. Accordingly, we name these peaks “spin-relaxation boom”. The total relaxation is a simple sum of contributions from LA and TA phonons.

In most cases, “spin relaxation boom” caused by LA phonons is the stronger peak, as shown in Figs 2 and 4(a). However, in other regimes (such as in a high magnetic field when phonon bottleneck effect^32^,^33^ starts to affect electron-phonon coupling strength), “spin relaxation boom” caused by TA phonons can be dominant, as shown in Fig. 4(b). Experimentally, “spin relaxation boom” caused by TA phonons may be easier to observe since it happens at a lower moving speed. By using the interference of two orthogonal SAW beams in GaAs^15^, for example, the moving speed of the electrons can reach 4.14 × 10^3^ m/s, faster than the speed of bulk TA phonons in GaAs. This would allow direct observation of “spin relaxation boom” within current experimental technology.

**Figure 5:**
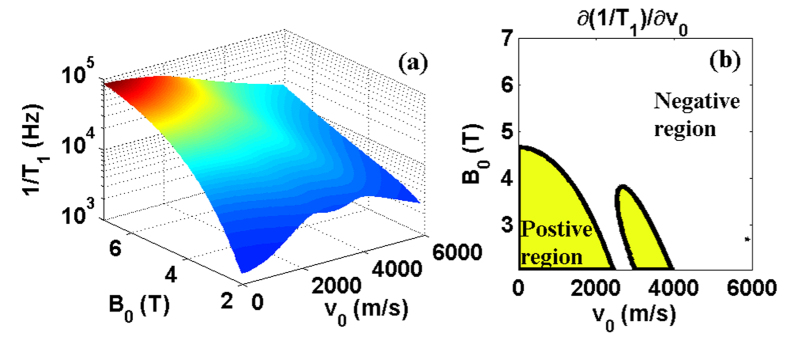
(**a**) Spin relaxation rate 1/*T*_1_ as a function of magnetic field *B* and moving speed of the quantum dot *v*_0_. (**b**) Partial derivative of 1/*T*_1_ with respect to *v*_0_. The white and the yellow regions indicate the partial derivative is below and above zero respectively. The parameters are chosen as *ω*_*d*_ = 1.1 meV, *d* = 20 nm, *ϕ*_*v*_ = 0.

One implication of the spin relaxation booms presented above is that a spin qubit can relax slower when the QD moves faster, as evidenced by the curves in Fig. 4. In Fig. 5(a) we give a more comprehensive view of this velocity dependence, plotting the spin relaxation rate as a function of the moving velocity *v*_0_ and the magnetic field *B*_0_. When the external magnetic field is relatively weak (e.g., *B*_0_ ≈ 2 T), the relaxation rate increases with the moving velocity. But when the external magnetic field is strong (e.g., *B*_0_ > 5 T), the relaxation rate becomes a decreasing function of the moving velocity. This somewhat counterintuitive feature is another consequence of the Doppler effect, where the Doppler-shifted frequency *w*_*z*_ for the emitted phonon depends on both the magnetic field *B*_0_ and the moving velocity *v*_0_. When the moving is fast, the Doppler effect is strong, shifting the phonon frequency into the range where phonon bottleneck effect suppresses electron-phonon interaction. The “shifted frequency” *w*_*z*_ also depends on the magnetic field *B*_0_. In a strong field, the Zeeman frequency *ω*_*Z*_ is already close to the bottleneck regime. It is thus much easier for Doppler effect to shift the frequency higher and suppress the phonon coupling.

Our observation here indicates that a moving spin qubit could have a lower relaxation rate than a static spin qubit. While “motional narrowing” is a common occurrence in spin resonance experiments^34^, suppressing decoherence by moving a spin qubit faster in a nanostructure setting is still an intriguing proposition. In Fig. 5(b), we plot the partial derivative of 1/*T*_1_ with respect to *v*_0_. Here the white region has 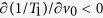, indicating where spin relaxation can be suppressed by increasing the moving velocity. On the other hand, in the yellow region 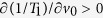, and relaxation becomes faster when the spin qubit moves faster. In short, the motional narrowing effect here shows the possibility of coherence-preserving transportation of a spin qubit. This motional narrowing would be effective for coherence if the regime of relaxation limited coherence is reached.

**Figure 6:**
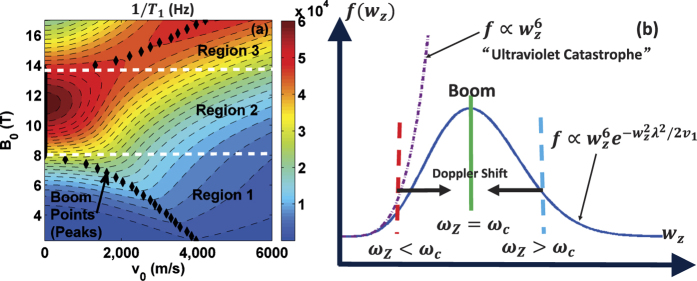
The left panel (**a**) is a contour plot for spin relaxation rate for deformation potential as a function of *B*_0_ and *v*_0_. The black points show the spin relaxation boom points (peaks of 1/*T*_1_). The right panel (**b**) shows the Doppler-shift influence on spin relaxation boom.

Equations (5, 6, 7, 8) show that spin relaxation boom is affected by three major factors, the quantum dot confinement effect described by cutoff functions (7), the magnetic field *B*_0_, and the moving velocity *v*_0_. More specifically, the confinement parameters *d* and *λ* set an upper limit on the phonon frequency. The interaction between the electron and phonon of higher frequencies is suppressed by the phonon bottleneck effect^32^,^33^. In a static QD, the phonon frequency is completely determined by the Zeeman frequency *ω*_*Z*_, thus only a strong *B*_0_ can cause phonon bottleneck effect. In a moving QD, the Doppler-shifted phonon frequency *w*_*z*_ is also affected by *v*_0_. These influences can all be observed in Fig. 6. In region 1 of Fig. 6(a), *B*_0_ is low, and the Zeeman frequency *ω*_*Z*_ is smaller than a critical frequency 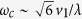. In this region a strong Doppler effect is needed to shift the phonon frequency higher into the boom region for spin relaxation rate to peak. When *B*_0_ is larger, *ω*_*Z*_ itself is already close to the boom region, so a relatively weak Doppler effect at low moving speed can already lead to spin relaxation boom. In the cases when the Zeeman frequency itself is already inside the boom region [region 2 in Fig. 6(a)], peak rate of spin relaxation can be observed even at *v*_0_ = 0. When we continue to increase *B*_0_, the Zeeman frequency already exceeds the boom region [region 3 in Fig. 6(a)], so that spin relaxation is suppressed. In this case, the Doppler effect may shift the phonon frequency downward to reveal the peak in relaxation rate.

In short, the spin relaxation boom appears when 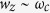. If the Zeeman frequency *ω*_*Z*_ itself is already close to *ω*_*c*_, the spin relaxation boom can be observed without Doppler shift. Otherwise a Doppler shift (either blue shift or red shift) can help reveal the spin relaxation boom, as is shown in Fig. 6(b).

## Discussion

Our study shows that in the supersonic regime for a moving spin qubit, only phonons emitted or absorbed in certain directions make notable contributions to qubit relaxation. As is shown in Fig. 3, the kernel function *f* for the spin relaxation rate 1*T*_1_ is non-zero only in certain directions in the *xy* plane. With this strong angular anisotropy, it is natural to consider whether we could eliminate spin relaxation by suppressing the electron-phonon interaction in certain directions. For example, if a phonon cavity is set up in a certain direction, the frequencies of phonon modes in that direction become discrete, making it possible to filter out important frequencies and to suppress electron-phonon interaction at those frequencies.

The narrowly directional phonon emission from the moving spin qubit may also point at a new source of non-classical phonons. Imagine a stream of excited spins moving supersonically in one direction. They would emit phonons in roughly two directions. If a phonon cavity is set up along one of those directions and is on resonance with the emitted phonons, it may be possible to create stimulated emission, even lasing, of phonons in that mode^35^. Furthermore, Fig. 6(b) shows that the spectral distribution of the phonons is concentrated around 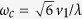. Consequently, no matter what the Zeeman frequency (red or blue) is (determined by the external *B*_0_ field), the actual emitted phonon tend to have a frequency around the boom region indicated by green line at the “spin relaxation boom”. Thus even before cavity selection the emitted phonons already have a narrow bandwidth because of the nature of the spin relaxation boom. We also note that in the electron-phonon interaction Hamiltonian, the coupling of the electron to the two phonon modes of the shock wave have equal strength. Our current approach treats these phonon modes separately within the limit of Fermi golden rule, thus we cannot tell whether there is quantum coherence between these modes, or whether the phonons may even be in a coherent superposition of these two modes. Investigating such coherent dynamics of the phonons would clearly be of interest to the realization of coherent phonon optics in semiconductor nanostructures.

The interesting features in phonon emission and spin relaxation we have explored here could be useful in monitoring and detecting the spin decoherence process. Conversely, knowing the phonon emission angle precisely may allow continuous monitoring of the environment, which could in turn provide more accurate information to possible feedback operations in a quantum feedback control^36^,^37^ or quantum state restoration^38^,^39^ scheme for the spin qubit. In an open quantum system, the information stored in the system constantly leaks into its environment. By measuring the environment in particular ways, however, the lost information could be partially or even fully regained. It is thus possible to restore a system to its initial state by monitoring the environment^39^. Our results about the angular distribution of phonon emission may provide a guidance on measuring the phonon environment: we can place the phonon detectors in selected directions [precisely predicted by equation (11)] since only phonons in those directions make significant contributions to spin relaxation.

In conclusion, we have studied decoherence of a moving spin qubit caused by phonon noise through SOI. The QD motion leads to Doppler shifts in the emitted/absorbed phonons by the moving spin qubit, which modify the spin relaxation rate. In particular, we find a “spin-relaxation boom” when the moving QD break the sound barriers, in analogy to the classical sonic boom. The occurrence of spin relaxation boom is determined by both magnetic field and moving velocity, and it implies the possibility of coherence-preserving transport of spin qubit by varying the moving velocity. The properties of the emitted phonons also undergo drastic changes as we vary the QD velocity. Specifically, when the moving velocity is larger than the speed of sound in the material, spin relaxation is dominated by phonon emission/absorption in certain directions. The physics here is similar to the phenomenon of classical Cherenkov radiation, with the emitted phonons highly directional and spectrally narrow. As such, moving excited spin qubits can also be thought of as a source of non-classical phonons.

## Methods

### Derivation of the effective Hamiltonian

An effective spin Hamiltonian, in which spin dynamics and orbital dynamics are decoupled, can be obtained by performing a Schrieffer-Wolff transformation to remove the SO coupling term in the full Hamiltonian^17^,^23^,^24^,^25^,^31^. Through a unitary transformation 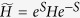, with *S* given by 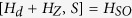, the SO coupling is removed to the first order. The spin Hamiltonian is then 
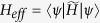
, where 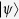 is the ground state of the orbital wave function. Following the approach used in refs 17,23, we obtain the effective Hamiltonian in equation (3), where Ω(*r*, *t*) originates from the electron-phonon interaction, and is given explicitly as


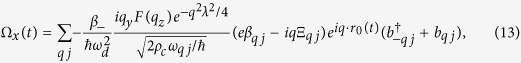



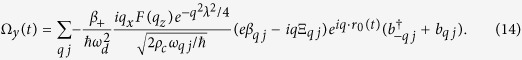


The expressions here are similar to the results in ref. 23, with an additional term 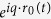 due to the motion of the quantum dot. This factor is also how Doppler effect is introduced into the dynamics of the moving spin qubit.

### Derivation of the spin relaxation rate

Given the effective Hamiltonian (3), the relaxation rate can be obtained within the Bloch-Redfield theory as 
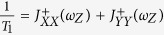
25, where *ω*_*Z*_ = *gμ*_*B*_/*ħ* is the Zeeman frequency. The tensors 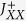 and 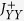 are correlations of the effective magnetic noise (from the phonons through the SO interaction),


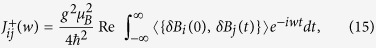


where


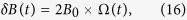


and Ω(*t*) is given in equation (13) and (14). These correlation functions are expressed in a rotated *XYZ* coordinate system, where the *Z* axis is along the direction of the applied field *B*_0_. Due to this rotation, a magnetic-angular dependence term *F*_*SO*_ appears in the expression of the relaxation rate^23^,^25^. With the magnetic noise from phonons, the relaxation rate takes the form


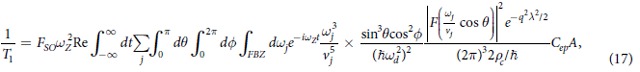


where


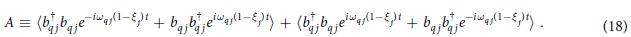


The time integral in equation (17) generates delta functions. But the integration over *dω*_*j*_ is in the first Brillouin zone, only the positive frequencies are taken into account, namely the integration only includes the case 1 − *ξ*_*j*_ > 0. Therefore, the condition 1 − *ξ*_*j*_ > 0 is combined with equation (8). Finally, we obtain the relaxation rate (5) and the kernel function (6). Compared with ref. 23, the phonon frequency here is shifted by the factor (1 − *ξ*_*j*_), which is a result of the Doppler effect. When the moving velocity approaches zero, the relaxation rate here reduces exactly to the result shown in ref. 23.

## Additional Information

**How to cite this article**: Zhao, X. *et al.* Doppler effect induced spin relaxation boom. *Sci. Rep.* **6**, 23169; doi: 10.1038/srep23169 (2016).
